# Suicidal jumper's fracture reduced with hyperextension and the joystick method: A case report

**DOI:** 10.1016/j.tcr.2021.100444

**Published:** 2021-02-20

**Authors:** Toru Matsugaki, Hideaki Shibata, Yuhei Esaki, Tsunemasa Matsubara, Ryota Takami

**Affiliations:** Department of Orthopaedic Surgery, Saiseikai Fukuoka General Hospital, 1-3-46 Tenjin, Chuo-Ku, Fukuoka city, Japan

**Keywords:** Suicidal jumper's fracture, Transverse sacral fracture, Hyperextension, Joystick method

## Abstract

Suicidal jumper's fractures are transversal fractures of the upper sacrum. The treatment for this type of fracture remains controversial. We present a case of a Roy-Camille type 2 suicidal jumper's fracture treated with reduction by hyperextension of the lumbosacral junction, the joystick method, and percutaneous fixation on the day of injury. After the operation, the sacral canal at the S2 level was enlarged and both lower extremities began to move gradually. At 19 days after the injury, direct decompression via sacral laminectomy was performed to promote further neurological improvement. At 10 months after the injury, cauda equina syndrome and radicular symptoms were completely resolved. Considering its minimal invasiveness, we recommend trying hyperextension and the joystick method to treat Roy-Camille type 2 suicidal jumper's fractures on the day of injury.

## Introduction

Suicidal jumper's fractures are transversal fractures of the upper sacrum [1]. Lumbosacral plexus injuries and cauda equina syndrome are present in nearly all cases and constitute major causes of late disability [2]. There is no consensus on how to treat this type of sacral fracture, especially on methods of reduction, and on the effect of reduction on neurological outcomes. We reduced a Roy-Camille type 2 suicidal jumper's fracture with hyperextension of the lumbosacral junction and the joystick method on the day of injury. We report this method and the patient's clinical course.

## Case report

**Fig. 1:**
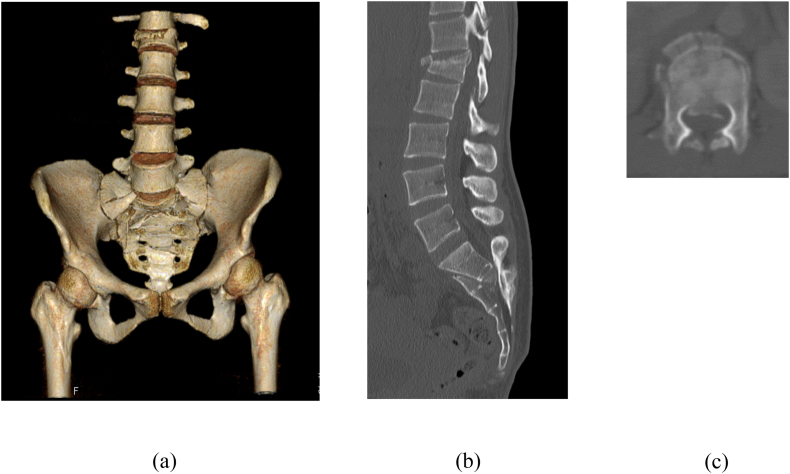
(a) Three-dimensional computed tomography (CT) showed an H-shaped Roy-Camille type II sacral fracture and a L1 burst fracture on admission. (b) Sagittal CT demonstrated the displaced fragment obstructing the sacral canal at the S2 level. (c) Axial CT showed 30% spinal canal compromise at the L1 level.

A 29-year-old woman fell from the fourth floor of an apartment building and was transported to the hospital by ambulance. On admission, her vital signs were stable. There were no voluntary contractions of the tibialis anterior, flexor halluces, or extensor hallucis muscles bilaterally. She had sensory deficits in the perineum. The anal reflex was absent. Computed tomography (CT) demonstrated a displaced, H-shaped Roy-Camille type 2 sacral fracture; an L1 burst fracture with 30% spinal canal compromise (Fig. 1); and bilateral calcaneal fractures. Soon after the initial assessment, she was transferred to the operating room for reduction of the sacral fracture.

**Fig. 2:**
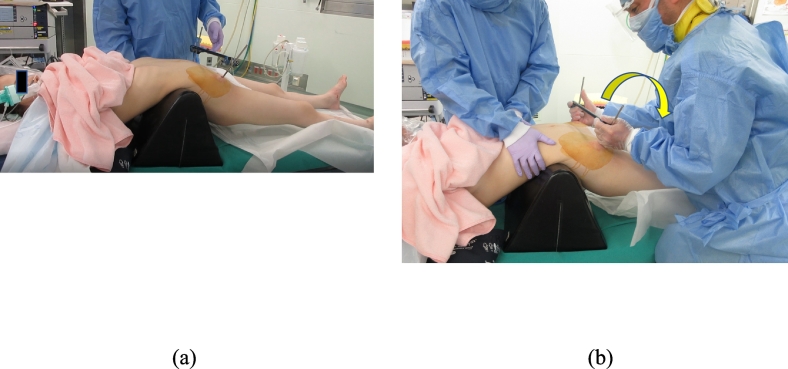
(a) Patient position during reduction of the pelvis. Hyperlordosis was induced by placing a triangular pillow under the lumbosacral junction. (b) The reduction force was applied in the direction of the arrow to hyperextend the lumbosacral junction.

**Fig. 3:**
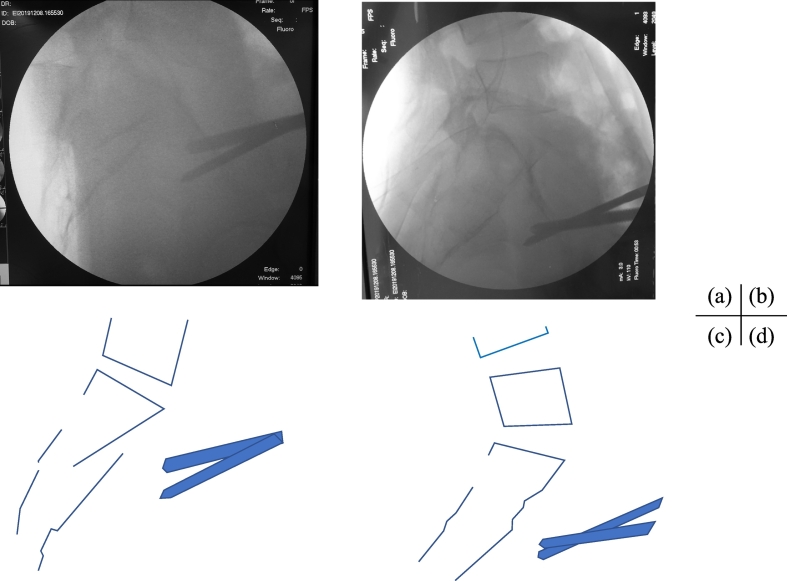
Intraoperative lateral fluoroscopic view (a) before and (b) after reduction. Schematic diagram (c) before and (d) after reduction. The angle of lordosis at the L5/S1 disc space after reduction was greater than the angle before reduction. The lower fragment moved dorsally and caudally with the half pins. The fracture was reduced with ligamentotaxis.

The patient was placed in the supine position on a transparent surgical table. Under general anesthesia, two 6-mm half pins were placed from the anterior inferior iliac spine to the posterior ilium on each side. They were connected with one bar and two clamps. Next, a triangular pillow was placed under the lumbosacral junction to induce hyperlordosis (Fig. 2a). At that time, the fracture had not been reduced yet (Fig. 3a). We applied rotational force to the ilium in the direction of extension with an external fixator on each side (Fig. 2b). A percutaneous screw was inserted after confirming that the fracture had been reduced appropriately with an image intensifier (Fig. 3b). Five days later, the screw was replaced with a long transiliac-transsacral screw at the same time as percutaneous fixation for the L1 burst fracture. During the initial emergent operation, we were only able to use a short screw. Although the right ankle and toe gradually began to move, direct decompression via sacral laminectomy was carried out at 19 days after the injury to promote further neurological improvement. Continuity of the sacral root near the fracture site was observed intraoperatively. Postoperatively, wheelchair transfers were started on the next day. Walking exercises were started 6 weeks later.

**Fig. 4:**
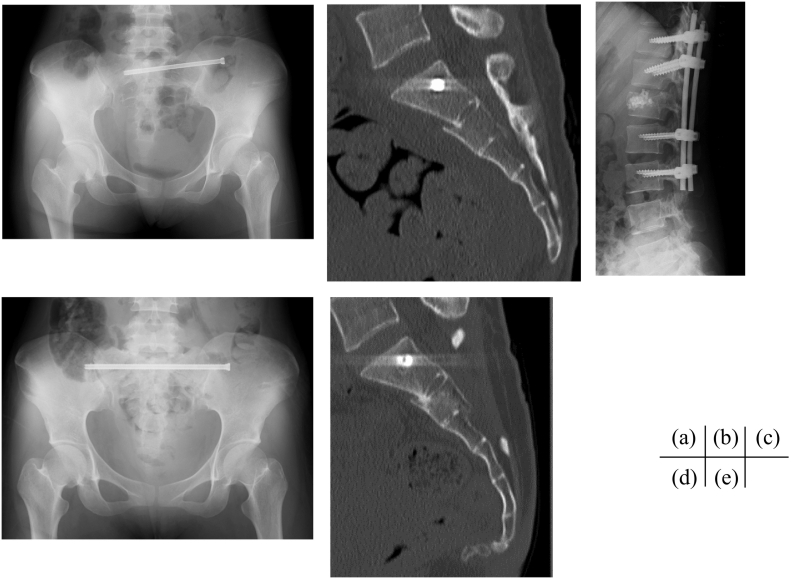
(a) Radiograph of the pelvis and (b) Sagittal computed tomography (CT) image after emergent surgery. The sacral canal at the S2 level was enlarged. (c) Radiograph of the lumbar spine after percutaneous fixation for the L1 burst fracture. (d) Radiograph and (e) CT image of the pelvis at 10 months after the injury.

Partial recovery of sphincter function was observed at 2 months after the injury. At 10 months after the injury, she could walk without assistance and voluntary contractions of the tibialis anterior and extensor hallucis muscles were normal bilaterally. She had full recovery of bowel and bladder function. The Majeed score was 95 points. CT showed bone union at the site of the sacral fracture and no obstruction of the sacral canal (Fig. 4c, d).

## Discussion

Roy-Camille type 2 suicidal jumper's fracture is a flexion fracture of the upper sacrum with posterior displacement of the superior fragment caused by a fall from a great height onto the lower extremities [1]. Displaced transverse sacral fractures result in cauda equina syndrome. The L5 and S1 nerve roots can be also injured as a result of vertical shear displacement of the sacrum [3]. Early realignment of the sacrum and decompression of the nerve roots is thought to provide the best possible environment for neurological recovery [[3], [4], [5]]. However, several days are often needed to optimize the patient's physiological status after severe blood loss and hemodynamic instability or severe concomitant injuries before open surgical treatment [3,4,6]. Therefore, closed reduction or percutaneous reduction is the optimal procedure to achieve indirect decompression on the day of admission.

Roy-Camille et al. reported that they applied heavy two-pole traction through the lower extremities to achieve closed reduction. However, reduction was not achieved [1]. Williams et al. applied bifemoral skeletal traction for reduction, but the effect of this reduction method is unclear because they added direct manipulation with iliac screws in case with insufficient reduction [7]. With these two methods, only a linear reduction force is applied; no rotational force is applied. Shieldhauer used a two-pin anterior external fixator for temporary reduction and stabilization as initial care [5]. However, the details of the method were not described. Ruatti et al. reported the hyperlordosis method, which involves placing folded sheets under the lumbosacral junction to induce ligamentotaxis [8]. However, in our patient, anatomical reduction was not obtained with the hyperlordosis method. The picture from the image intensifier after reduction, which achieved a rotational force with the external fixator, showed that the angle of lordosis at the L5/S1 disc space was greater than before reduction. Therefore, the anterior longitudinal ligament at L5/S1 was not sufficiently tense for reduction with just placing folded sheets or a pillow under the lumbosacral junction. However, when rotational force is applied to the ilium bilaterally by an external fixator, the anterior longitudinal ligament becomes tense enough to fix the upper fragment. If both ilia are rotated further, the fracture will be reduced as the lower fragment is pulled in the caudal and dorsal directions.

Full recovery of bowel and bladder function and muscle strength in both lower extremities occurred by 10 months after the injury. It is unclear whether this recovery was due to reduction or laminectomy. However, because neurological status improved before laminectomy and CT revealed an enlarged spinal canal at the S1/2 level after reduction, we believe that reduction contributed more to recovery than laminectomy. Hyperextension and the joystick method are noninvasive procedures that induced enough ligamentotaxis to reduce the fracture. We recommend trying it for reducing Roy-Camille type 2 suicidal jumper's fractures soon after the initial assessment and resuscitation.

## Declaration of competing interest

The authors declare that they have no conflicts of interest in connection with this paper. All authors confirm that they have no financial or personal relationships with other people or organizations that could inappropriately influence this work.
